# High throughput screen for the improvement of inducible promoters for tumor microenvironment cues

**DOI:** 10.1038/s41598-022-11021-1

**Published:** 2022-05-03

**Authors:** Omri Sharabi, Yariv Greenshpan, Noa Ofir, Aner Ottolenghi, Tamar Levi, Leonid Olender, Zachor Adler-Agmon, Angel Porgador, Roi Gazit

**Affiliations:** The Shraga Segal Department of Microbiology, Immunology, and Genetics, Faculty of Health Sciences, Ben-Gurion University of the Negev, Beer Sheva, Israel

**Keywords:** Cancer microenvironment, High-throughput screening, Genetic engineering

## Abstract

Cancer immunotherapies are highly potent and are gaining wide clinical usage. However, severe side effects require focusing effector immune cell activities on the tumor microenvironment (TME). We recently developed a chimeric antigen receptor tumor-induced vector (CARTIV), a synthetic promoter activated by TME factors. To improve CARTIV functions including background, activation levels, and synergism, we screened a library of promoters with variations in key positions. Here, we present a screening method involving turning ON/OFF stimulating TNFα and IFNγ cytokines, followed by sequential cell sorting. Sequencing of enriched promoters identified seventeen candidates, which were cloned and whose activities were then validated, leading to the identification of two CARTIVs with lower background and higher induction. We further combined a third hypoxia element with the two-factor CARTIV, demonstrating additional modular improvement. Our study presents a method of fine-tuning synthetic promoters for desired immunotherapy needs.

## Introduction

Immune cells are programmed to express effector genes when needed. Interestingly, few genes are transiently induced following activation by external ques^1^. Spatiotemporal control over gene expression may improve engineered immune cells.

### The need for inducible promoters

Immune therapies can eliminate cancerous cells within the body^2^. Effector immune cells that recognize and attack tumor antigens have already gained clinical approval for chimeric antigen receptor T-cells (CAR-T)^3^. However, the vigorous activities of CAR-T are also a risk factor^4^. CAR-T responds to even minute amounts of antigens and may cause severe cytokine storm syndrome, requiring additional regulation of the engineered cells^5^. To reduce life-threatening side effects, CAR-Ts are being developed with an intrinsic self-destruction system^6^, or with ON/OFF switches^7,8^. Improving the spatiotemporal regulation of CAR-T and other types of engineered immune effectors will create safer applications of such robust tumor-killing cells^9^.

### Chimeric antigen receptor tumor-induced vector (CARTIV)

We recently published a novel approach to regulate effector immune cells using promoters that are inducible by the tumor microenvironment (TME)^10^. CARTIVs are built by combining binding sites that respond to factors present within the TME. Since tumor microenvironments diverge from normal tissues^11^, we thought to utilize TME factors as inducers for effector immune cells to act against cancerous cells, and spare normal tissues^10^. This is somewhat recapitulating the endogenous immune system control of inducing activity within inflammatory sites in vivo, thus reducing collateral damage to healthy tissues. Our initial CARTIV promoters demonstrated specific responses to some of the major inflammatory cytokines, such as gamma-interferon (IFNγ) and tumor necrosis factor-alpha (TNFα), together with hypoxia. Importantly, numerous studies have attempted to develop such tumor-specific promoters, taking different approaches^12–15^. We developed the CARTIV approach by combining several minimal binding sites and defined spacers, which resulted in nontrivial findings regarding the number of sites and their relative positions in the promoter^10^. Our CARTIV promoters are rather short, ranging between 200 and 300 bases only, and modular, to allow further adjustments and specificity to TME factors. Notably, the first study used basic elements that are not necessarily the best binding sites for background expression or maximal fold induction, creating interest in further improvements.

### Rationale for screening promoters with variations on a theme

Promoters have "canonical" binding sites of transcription factors, allowing the synthetic design of artificial sequences that may have specific gene expression potency^16^. Even with advanced protein structure prediction^17^, it is not easy to predict the complex interactions of several factors with DNA and their cumulative induced transcription. Natural promoters have evolved to provide adequate induction of genes, with divergent response time and magnitude. Changes in the sequences of CARTIV promoters will lead to changes in responses to given stimuli. Specifically, changing only several defined key positions within a given promoter may result in a stronger or more specific response, which is of great interest for clinical applications. Optimization of sequences through screening of libraries is well established^18–20^, with a focus on key positions being possible^21^. For example, a random DNA sequence of 12 bases has a complexity of 4^12^ (over 16 million sequences); rational focus on key positions within binding sites may allow manageable permutations for even a 100-base-long promoter.

We choose lentivirus as a transfection vector since it is the accepted practice of CAR delivery into T cells, both in research and in the clinic^22,23^. While chromosomal positional effects may have an impact on gene expression; we transduce lentiviruses as batch and study the population of cells, not clones. Importantly, several recent publications scanned promoter libraries with lentivirus, successfully^24,25^. Hence, we choose to use the same vector as our recent publication^10^.

### Structure of canonical CARTIV promoter elements

The original CARTIV promoters^10^ are based on canonical binding sites. IFNγ signaling through STAT1 includes binding with IFNγ activation site GAS, consensus sequence TTCCNGGAA^26^. TNFα signaling through NF-kB binds with p50, consensus sequence GGRRRTTYYC, where R is A or G, and Y is C or T^27^. The hypoxia response elements may include HIF consensus sequence RCGTG^28^. We simplified these core sequences and added rationally designed repeats and spacers^10^. Notably, we already achieved nontrivial results regarding the order by which basic elements were assembled when combining hypoxia plus GAS plus kB elements^10^. Improving CARTIV, or any inducible promoter requires screening of focused libraries by functional selection for the desired phenotype. CARTIV promoters already show a good response to TME, but may benefit from additional improvements for specific expression levels.

In this study, we aimed to improve CARTIV promoters by functional screening of variations on a theme. Improved promoters may provide nontrivial properties such as robust induction, low background, or synergism. First, we focused on critical nucleotides predicted to allow for variations without losing transcription-factor binding. We constructed libraries limited to less than 10^5^ complexity, transduced cells in culture, and screened them through subsequent rounds of sorting with and without activating cytokine. Sequencing identified multiple enriched variants of interest. We further validated some 15 candidates and found improved properties over the original CARTIV promoters. Importantly, we noted that exceptionally high induction or very low background may require different promoter sequences. We also demonstrated that variable nucleotides may have both direct and indirect interactions with their cognate transcription factors. Taken together, we present a method for the functional screening of hundreds of thousands of variant promoters and the identification of improved functionalities.

## Material and methods

### Library cloning

The library sequence used:

cccggtcgcactagttctagaAYTTCCSGGAARTAGGGTGGGCAAGTAYTTCCSGGAARTtctagaGGRRRTTYYCGGGGACTTTCCGGRRRTTYYCtctagaTATTAAGGTGACGCGTGTGGCCTCGAACACCGAGCGACCCTGCAGCGACCCGCTTAAAAgcggccgccATG.

The oligo, 4 nmol, was ordered from Integrated DNA Technologies (Coralville, IA). The library was amplified (15 cycles) using PrimeStar© max (Takara Bio, CA) with primers containing SpeI and MluI restriction enzyme sites. DNA fragments were resolved on agarose gels, extracted, digested, and cloned into pHAGE2 plasmids containing fluorescent reporters. Plasmids were electroporated into NEB 10-beta cells that were grown overnight in 300 ml LB and then extracted using a midiprep kit (Macherey–Nagel, Duren, Germany).

### Tissue culture and cytokines

HEK293T cells were grown in DMEM containing 10% serum, Pen-Strep, HEPES, l-glutamine, non-essential amino acids, and sodium pyruvate (Biological Industries, Beit Haemek, Israel). Cells were grown at 37 °C in a humidified 5% CO_2_ incubator. Human recombinant IFNγ and TNFα with activities of 2 × 10^7^ Units/mg were purchased from PeproTech (Rehovot, Israel). Standard lentivirus production was as reported^10^.

### Primary human T-cell isolation, culture and transduction

Primary T cell culture were established, maintained and transduced as described elsewhere^10^. Briefly, human PBMCs were isolated from a healthy consenting donor’s blood by Ficoll gradient. Cells were activated using soluble anti human CD3 OKT3 (biolegend, CA) for 48 h and transduced using RetroNectin (TAKARA BIO, Japan) coated plates according to the manufacturer recommendations. Two days following transduction culture was stained for CD3 to validate T cell expansion.

### CARTIV promoter activity assay

HEK293T cells were plated at 1 × 10^5^ cells per well in 96-well flat-bottomed plates. Cytokines were added to final concentrations of 500 U/ml. In experiments involving hypoxia, cells were cultured in a hypoxic chamber with a gas mixture of 5% CO_2_, 0.3% O_2_ and 94.7% N_2_ at 20 l/min for 3–5 min, and then sealed and placed at 37 °C for 16–20 h before analysis. Cells were harvested, suspended with DAPI 1 µg/ml and measured by FACS using a Beckman Coulter® Gallios™, Cytoflex or CytoFlexS flow cytometers. Data were analyzed using Kaluza™ or the CytExpert software.

### CARTIV library sorting

HEK293T cells were plated at 3 × 10^6^/10 cm plate. Cells were transduced the following day at an MOI of < 0.3 (calibrated to have fewer than 30% positive cells to minimize double transduction^25^). Cells were harvested and re-plated for activation at least 72 h post-transduction. Cells were sorted by fluorescent-reporter expression using a FACS aria III (BD) cell sorter for each of the three rounds of selection.

### Library sequencing

Genomic DNA was extracted and promoters were amplified by PCR for 22 cycles and sent for next-generation sequencing (NGS) at HyLabs (Rehovot, IL). The average number of sequences per sample was 22,368. BioSample metadata are available at the NCBI BioSample database under accession SAMN 21397074: CARTIV G1K1 Library (TaxID: 9606).

### Potency index

Potency was calculated using the equation $f(x)=\frac{x(GK)}{bg\left(GK\right)}-\frac{x}{bg}+\frac{x(GK)}{xG+xK}$*,* where ‘x’ is the normalized GeoMean, ‘G’ and ‘K’ are IFNγ and TNFα treatments, respectively, and ‘bg’ is background GeoMean. When no G or K present, the GeoMean is of cells expressing miniTK alone.

### Statistics

FACS data are shown as mean ± SD. Data are representative of at least three independent experiments unless otherwise noted. A two-tailed T-test was performed, with p < 0.05 considered significant.

## Results

### CARTIV promoter library

**Figure 1:**
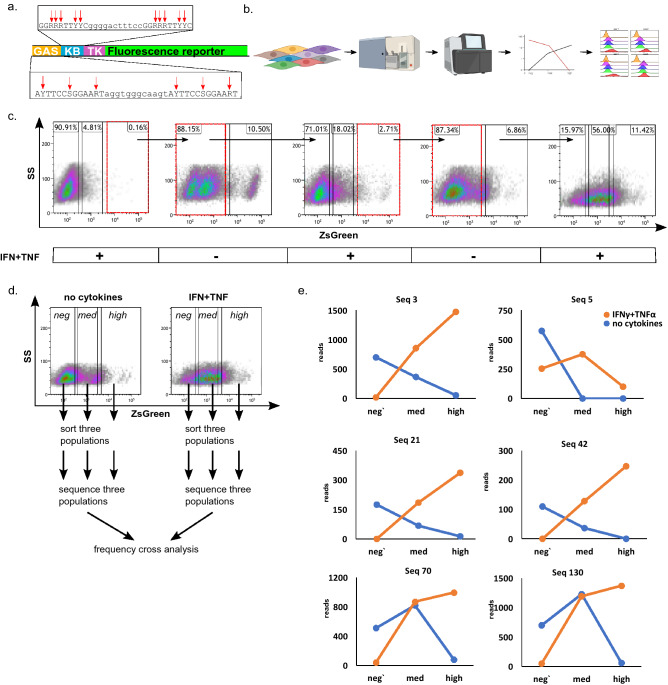
The GK CARTIV promoter library showing increments for responsive promoters following three sorting rounds. (**a**) The basic structure of the G1K06 CARTIV promoter and the variable elements used to construct the CARTIV promoter library. Red arrows indicate variable nucleotide position. Y = C/T, S = G/C, R = A/G. (**b**) An overview of the steps applied to the library screen. (**c**) FACS plots of HEK293T cells infected using lentiviruses with ZsGreen under the control of the CARTIV promoter library, showing three consecutive rounds of activation and relaxation. Red boxes and arrows show the sorted population in the specific selection round, 72 h post-infection. Cells were treated with 500 U/ml of IFNγ and TNFα for 48 h, then harvested and processed for cell sorting. Data are single-discriminated. (**d**) FACS plots of HEK293T cells after three consecutive rounds of positive and negative selection, showing data and gating for the six-cell population extracted for sequencing and frequency cross-analysis. Data are single-discriminated and DAPI-negative. (**e**) Frequency cross analysis for six representative promotors selected for the functional studies. Cut-off for reading number was 150; only sequences showing “x” trend were selected for cloning.

CARTIV promoters were initially designed by combining multiple binding motifs and linkers^10^, but were not optimized. In order to improve functional parameters of CARTIV promoters, such as reduced background, increased induction, and synergism, we designed a library based on CARTIV promoter G1K0.6 (Fig. 1a). CARTIV promoter response elements (CPREs) for IFNγ (GCPRE) and TNFα (KCPRE) were adapted from published binding motifs^26,27,29^. A library with 16 variable positions provided a reasonable complexity of 65,536 sequences. For the GCPRE element, we employed the YTTCCSGGAAR sequence (where Y = C/T, S = C/G, and R = A/G). The primary design and the 3′-positions were selected based on the IFNγ-PRE consensus^30^. We introduced a tandem repeat of this core into the template, separated by a linker AGGGTGGGCAAGT (Fig. 1a). For the KCPRE element, we employed the GGRRRTTYYC core^26,27,29^ separated by a linker GGGGACTTTCC (Fig. 1a). DNA oligos with libraries also included a minimal herpes virus thymidine kinase (mini TK)^31^. We amplified and cloned them into a lenti-vector (Supplementary Fig. 1), as before^10^, producing libraries of CARTIV promoters with variable key nucleotides.

### Functional screening resulted in improved CARTIV properties

To test whether we could find improved sequences, we screened the libraries by functional activation and sorting (Fig. 1b). Three days after transfection of the HEK293 cells with library LVs, we supplemented cell medium with IFNγ and TNFα, and sorted the fraction of responsive cells forty-eight hours later (Fig. 1c). The fraction of positive cells was kept low to avoid multiple integration into single cells. Sorted cells were grown for at least 72 h without cytokines, expanded and sorted for the fraction of cells showing reduced expression (Fig. 1c). Notably, a substantial fraction continued to show high expression, suggesting an undesired slow OFF rate. We collected both low-expressing cells and those returning to background levels. We repeated this ON/OFF sorting twice more (Fig. 1c), resulting in substantially more positively-induced cells and better decrease rates than primary populations. Finally, we split the double-sorted cells for growth with or without cytokines. In the last sort, we collected six populations according to "low", "medium", and "high" expression from the activated and non-activated groups (Fig. 1d), identifying cells with desired ON/OFF switching and coupled controls. Integrated CARTIVs were PCR-amplified and sequenced.

To count how many times each sequence appeared in each treatment, we used the DADA2 algorithm to distinguish single-base resolution^32^. Counts resulted in a total of 497 unique enriched sequences from the library (Supplementary Table 1). Sequences with fewer than 150 reads from all six samples were excluded, leaving 161 enriched candidates. Sequences were ranked according to their abundance. We chose sequences that counted fewer reads in the negative fraction and more reads in the medium and high fractions of the activated samples, or an inverse trend in the non-activated samples (Fig. 1e). Following this logic, we selected 17 top-ranked candidates (Supplementary Table 2). Thus, functional screening of the CARTIV library identified a manageable list of variants.

### Enriched sequences reveal variability of key nucleotides

**Figure 2:**
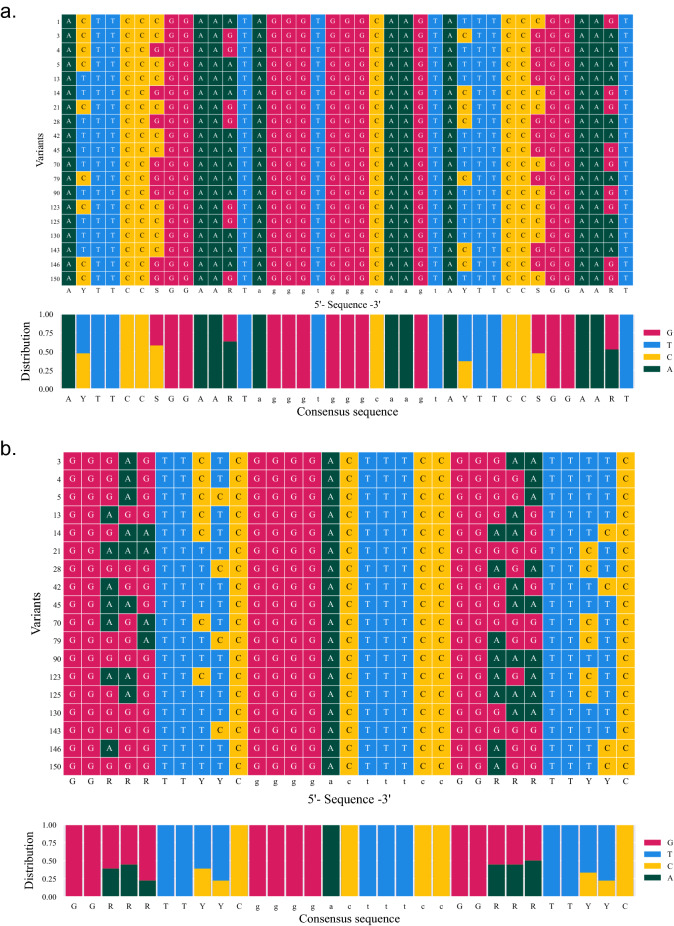
The selected GK CARTIV promoter library shows no strong tendency for a specific position. (**a**) Multiple sequence alignment of GAS elements. (**b**) MSA of KB elements. Each row represents a sequence derived from the bioinformatics analysis. A distribution analysis between 0 to 1 is shown below A and B.

**Figure 3:**
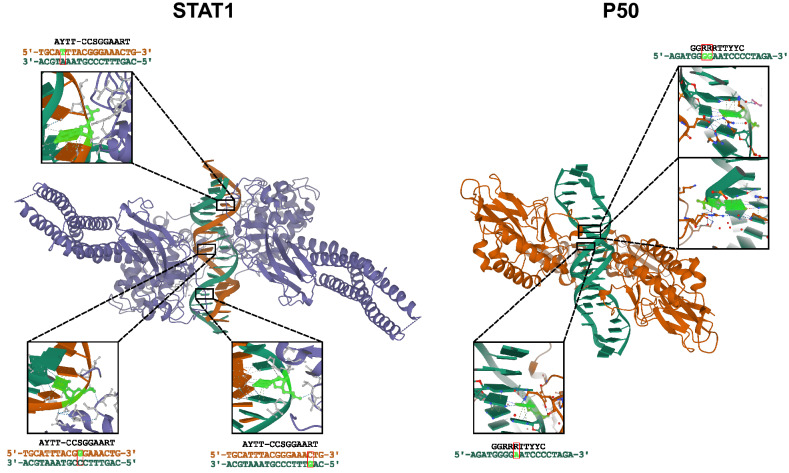
DNA in the variable positions in the CARTIV library interact with P50 and STAT1 by the phosphate backbone structures of STAT1 and P50 crystalized with a consensus DNA sequence. Left panel: the crystal structure of STAT1 together with STAT1 binding sequence, in orange the 5′ DNA strand, in dark green the 3′ DNA strand. In bright green, the nucleotide interacting with the protein, marked on the DNA sequence, is the relevant nucleotide pair, indicating the interacting nucleotide. The right panel shows the crystal structure of P50 with a binding sequence; the 5′ DNA strand is shown in dark green. The nucleotide interacting with the protein marking the relevant nucleotide pair on the DNA sequence and indicating the interacting nucleotide is shown in bright green.

Next, we wanted to check if any position held profound bias for specific nucleotides. Surprisingly, when examining the selected sequences by multiple sequence alignment, no single variable position was "locked" to a specific nucleotide. In the GAS element, the ratio of the variable positions ranged from 0.36 to 0.52 and averaged 0.43, while in the kappa element, variable bases ranged from 0.22 to 0.5 and averaged 0.35 (Fig. 2). The lack of profound bias suggests a high degree of freedom for these positions. In order to better realize the significance of the identified variable nucleotides, we examined the crystal structures of STAT1 (PDB 1BF5) or P50 (PDB 1SVC) with the relevant motif sequences (Fig. 3). In both STAT1 and P50 we noted that the protein-DNA interactions are achieved mostly through the DNA phosphate backbone, and not directly over the nitrogenous base of the variable bases (Fig. 3). Hence, structural analysis supports the high degree of freedom found in these specific nucleotides, suggesting no strict exclusions but rather good impartiality, allowing fine-tuning of promoters by variation.

### Nontrivial functionalities of selected promoters

**Figure 4:**
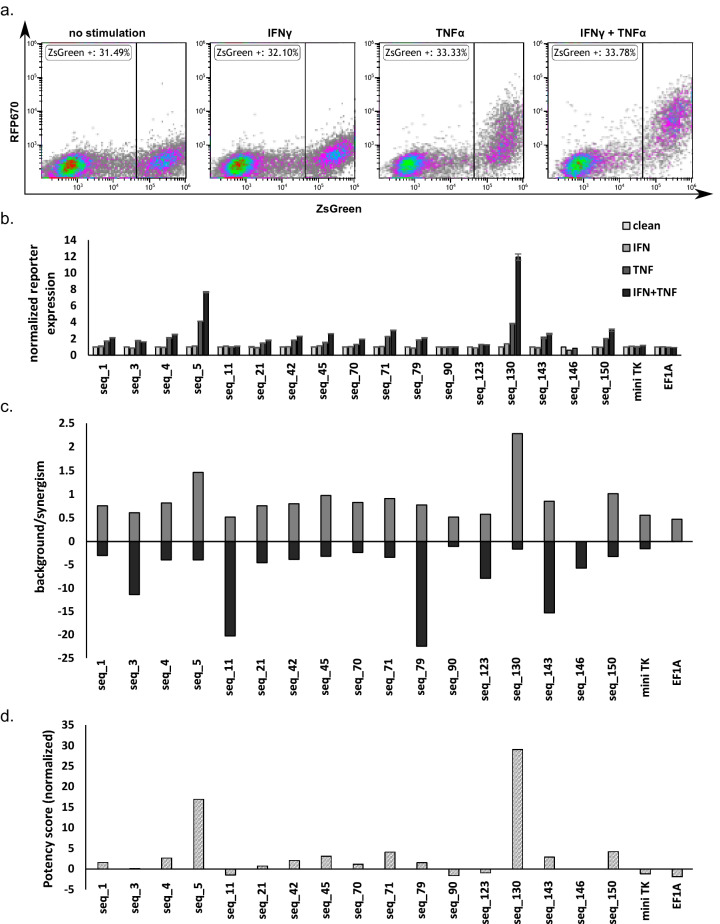
Promoters 5 and 130 show a robust and synergistic response following IFNγ and TNFα activation. (**a**) Representative FACS plots from seq 130. Cells were single-discriminated and gated on DAPI- (**b**) Normalized reporter expression. Each clone was treated with IFNγ and TNFα. The reporter’s geometric mean fluorescent intensity (MFI) of each clone was divided by the response of the non-treated cells. (**c**) Synergism was calculated by dividing the MFI of cells stimulated with IFNγ and TNFα divided by the sum MFI of cells stimulated with IFNγ or TNFα. background levels (solid gray bars) were calculated by dividing the MFI of transduced cells by the MFI of non-infected cells in the same well. (**d**) Potency score (dashed bars). Data from one of two experiments are shown.

To test for the functional improvement of identified enriched sequences, we aimed for independent validation. All 15 selected variants were synthesized de novo, cloned, produced individually in lentiviruses and transduced separately into fresh HEK293 cells. After expansion, cells were split into separate wells for activation by either IFNγ or TNFα, or a combination of both (500 U/ml). FACS analysis measured induction of the fluorescent reporter (Fig. 4a, and Supplementary Figs. 2 and 3). Notably, little response was elicited by IFNγ alone, while most sequences showed a significant response to TNFα and even higher induction by a combination of IFNγ and TNFα (Fig. 4b). Sequences 79 and 143 exhibited the highest overall response by simple gross analysis, while sequences 4, 5, 11, and 130 also showed a substantial induction (Supplementary Figs. 2 and 3). Analysis normalizing reporter expression to the no-cytokine baseline showed that sequences 5 and 130 had the highest specific response (7.66- and 11.92-fold increase, respectively). Seq 5 and Seq 130 also showed the highest specific response to TNFα alone (4.12- and 3.83-fold increase, respectively). Intriguingly, IFNγ did not elicit a very strong response by itself but showed major contribution when coupled with TNFα. Thus, we wanted to find out whether the effect of cytokine combination was additive or synergistic. We divided the response of the promoter to the combination of cytokines with the sum of responses to IFNγ and to TNFα (MFI_gk_/(MFI_g_ + MFI_k_)). By this analysis, the combination of cytokines had the most synergistic effect in sequences 5 and 130 (1.46 and 2.28, respectively, Fig. 4c). Out of the 15 enriched sequences, we found independent validation for improved induction and synergism, with two leading hits for further use.

An important parameter for CARTIV promoters is their background level, i.e., expression without cytokines. To determine background, we measured the fluorescence levels of cells transduced with vectors having the miniTK promoter only, and compared them with the selected CARTIVs without cytokines. Data indicated that sequences 90 and 130 had the lowest background (1.04 and 1.63, respectively). In contrast, sequences 11, 79, and 143 had the highest background (5.24, 12.38, and 15.29). In addition, other variants showed low background, among them sequence 5 (Fig. 4d and Supplementary Figs. 3 and 4). This suggests that variations of key nucleotides may provide different levels of background or basal leakiness.

In order to logically score the overall functionality of the selected promoters, we calculated a "Potency index" (see “Methods”), taking into account activation levels, background levels, and synergism. In accordance with the above-mentioned activation data, Seq 130 and Seq 5 appeared to be the most potent (11.38 and 6.96 scores, respectively). In contrast, some promoters achieved a score close to zero, including the controls, miniTK, and the ef1α promoters (Fig. 4d). Thus, promoters achieving high potency scores have high activation levels and high synergism levels between their promoter response elements (PREs) and low background levels. This index further suggests nontrivial improvements following screening through random changes of nucleotides within CARTIV promoters.

**Figure 5:**
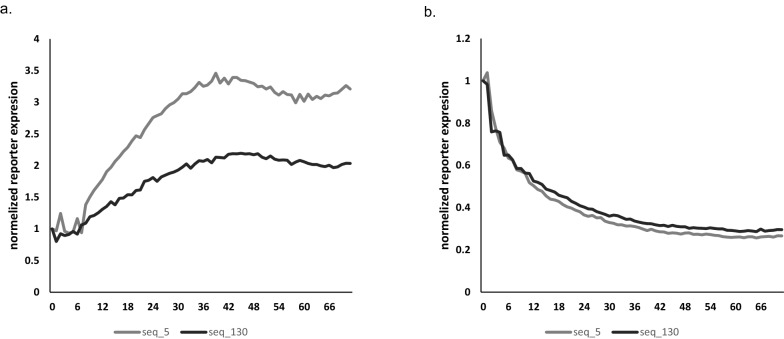
Sequence 5 displays better activation kinetics. (**a**) Activation rate. Y-axis: normalized RFP670 expression; X-axis: time. The time interval is 1 h. (**b**) Deactivation rate. Medium was replaced with fresh medium without cytokines at time zero.

Next, we wanted to test the time it takes to turn the new variant promoters on and off. The leading hits, Seq 5 and Seq 130, were tested for their kinetics. Transduced HEK293 cells were activated by IFNγ and TNFα (500 U/mL each) and tracked using a lionheart fx automated microscope. Seq 5 showed a faster ON rate (Fig. 5a). Next, we withdrew cytokines and followed the cells. As seen in Fig. 5b, the “OFF” rate was very similar for Seq 5 and Seq 130. Taken together, variations of key nucleotides change not only expression levels, but also the relative kinetics of induction.

### Additional hypoxia PRE can further enhance the activity of selected promoters

**Figure 6:**
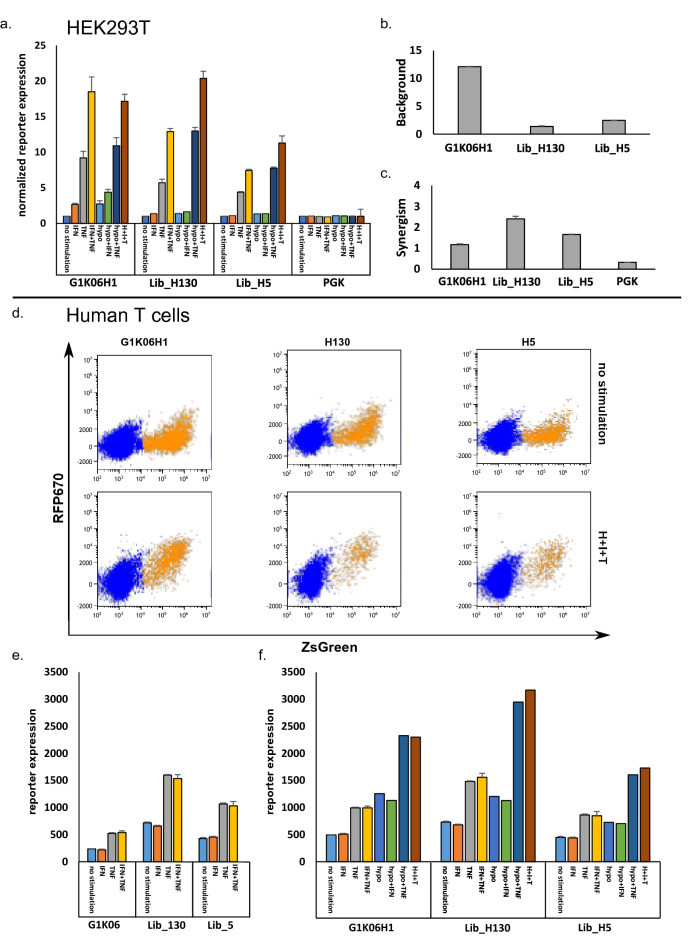
Adding complexity to the G1K06-5/130 promoters retain the response to external stimuli. (**a**) Hypoxia element was added to the G1K06 library backbone. The normalized expression of RFP670 in ZsGreen-positive cells shows the average of triplicates. Error bars indicate standard deviation. (**b**, **c**) The background and synergism of the indicated promoters were calculated as described in materials and methods. (**d**) Representative FACS plots of the 3 promoters. Top panel is no stimulation while the bottom panel is IFNγ, TNFα, and Hypoxia (**e**, **f**). Promoters were tested in human primary T-cells. Data is single discriminated DAPI− and ZsGreen+. Error bars indicate the standard deviation of triplicates.

In a previous study, we investigated the contribution of hypoxia PRE (HCPRE) by adding it to CARTIV promoters^10^. Since Seq 130 and Seq 5 had the best potency scores of the G1K06 variants tested, we sought to combine them with HCPRE. Promoters HG1K06-130 (H130) and HG1K06-5 (H5) were cloned and transduced into fresh cells. After expansion, the cells were tested without cytokines, with IFNγ, with TNFα, or with both, and subjected to hypoxic conditions for the last 18 h before reading signal intensity. In agreement with previous experiments, the combination of IFNγ and TNFα resulted in high induction. The hypoxia HCPRE further enhanced the reporter levels (Fig. 6a). Analyzing for specific expression (normalizing to background) revealed that H130 had higher-fold induction than both the original G1K06H1 and H5 (Fig. 6a). Library hits retained a better background and synergism indexes compared to the original promoter (Fig. 6b,c).

Furthermore, we evaluated the activity of the promoters in human primary T-cells. Both the original 130 and 5 as well as H130 and H5 promoters showed a response in human primary T-cells. As previously described, the TNFα and hypoxia stimuli were responsive while the IFNγ was not probably due to autocrine IFNγ secretion^10^. Notably, both 130 and H130 had higher maximum induction compared to the original G1K06 and G1K06H1 respectively (Fig. 6d–f).

Hence, the modularity of CARTIV promoters is sustained with improved sequences, allowing for further addition of response elements that show higher levels of expression. Our improved CARTIV promoters retain the modularity and possible combination with additional PRE for more TME factors.

## Discussion

### Focus on the tumor

In this study, we functionally screened de novo variations of synthetic promoters. A series of FACS sorting with and without stimulation (ON and OFF states) identified numerous candidates that were validated independently, revealing nontrivial combinatorial effects of key nucleotides. We demonstrate improvements including low background and high fold activation in response to two factors characteristic of TME. Moreover, we show that these improved promoters can further benefit from the addition of a third hypoxia element, demonstrating the modularity of the CARTIV design.

### CARTIV elements—the basis for variations

Our first line of CARTIV showed good induction by a combination of TME factors, such as TNFα, IFNγ, and hypoxia^10^; however, activation by IFNγ alone was relatively low. The basic GAS PRE element has the typical sequence for STAT binding^26^. Variations of selected nucleotides (Figs. 3, 4) showed a modest increase of IFNγ alone, and a more substantial synergistic induction together with TNFα. These data suggest some freedom of these nucleotides, not abrogating the interactions with STAT proteins but rather fine-tuning the CARTIV activity. Importantly, in this study we focused on changes within binding sites, while the synergistic activities may be further modified according to the linkers, opening the opportunity for additional improvements.

The STAT proteins contact the GAS DNA sequences in a 15-bp region^26,33,34^. According to Chen et al*.*^34^, the optimal DNA binding for STAT1 was suggested as AHTTCCSGGAAD (or explicitly, A[A/C/T] TTCC[C/G]GGAA[G/A/T]TG). For library construction we used AYTTCCSGGAARTG (Fig. 1a). In our 15 selected promoters we saw no profound bias towards a single nucleotide at any of the variable positions (Fig. 3). Importantly, Seq 130 that showed the best synergism results from having only 2-base difference from Seq 5, and no obvious overall difference from all other 15 CARTIV promoters (Fig. 3). Therefore, our data suggest a nontrivial effect of specific nucleotide variations on the overall activity, and the synergistic effect of the GAS portion with the CARTIV promoter.

The CARTIV kappa element was based on the sequence GGRRRTTYYC^27^. Chen et al*.* published a similar core sequence of GGGRNWTTCC^35^. In our 15 sequences, we found sequences starting with GGGG associated with p50, and others starting with GGAA associated with RELA^27^. Interestingly, Seq 130 has GGGGG in the first kappa element and GGGAA in the second. This suggests binding of RELA-p50 to the first site and RELA-RELA to the second site. On the other hand, Seq 5 has GGGAG and GGGGA, suggesting that both may bias for RELA-p50 and not for RELA-RELA. Activation by TNFα only was similar between Seq 5 and Seq 130, but synergism was better with Seq 130, possibly due to the heterogeneous usage of the binding dimers. Our data suggest that kappa PRE may respond promiscuously to p50 and/or RELA.

### Nontrivial functions of selected CARTIV promoters

We have previously shown that it is possible to increase expression levels by increasing repeats of elements (e.g., G1K1 < G2K2 < G3K3). However, this may cost substantial background expression and reduction in synergism^10^. One of the major limitations of CAR-T treatments in solid tumors is the “on target, off-tumor” toxicity^4,36,37^. Here, a library screen identified variants with nontrivial reduced background and increased synergism (Fig. 4). The method described here is not a one-dimension promoter improvement. Indeed, we yielded either reduction in background, improved synergism, and/or overall expression (Fig. 6). Such improvements may provide for sufficient CAR expression at the TME, sparing normal healthy tissues. Variations on the theme of modular CARTIV promoters offer fine-tuning and adjustments of CAR or other immunotherapies where spatiotemporal control is needed^9,38^.

Using lentiviruses for screening of promoter libraries is non-trivial. While we cannot rule out positional effects, the fact that we see reproducible changes of activity between promoters with only a few mutations, in independent experiments having independent batch transductions, strongly suggests that our CARTIV promoters are the main effector on reporter expression.

### Structural consideration of variable bases

According to structural data, the selected variable positions are within the DNA–protein interactions (Fig. 4). The high degree of freedom noted in these positions (Fig. 3) implies that when designing a promotor sequence, it is advisable to examine all positions that interact or are predicted to interact with the protein by the phosphate backbone rather than the nitrogenous base. Fang et al*.* showed that a single SNP in a DNA binding recognition site can influence transcription factor binding, thereby affecting gene regulation^39^. Thus, our engineering of synthetic promoters is also relevant for natural variations among humans. One may assume low or no tolerance for variability where a nitrogenous base interacts directly with a transcription factor. Interestingly, Le et al*.* demonstrated that dinucleotides flanking the core promoter sequence can contribute significantly to transcription factor binding^40^, thus adding fine-tuning of activities. Nevertheless, as we have demonstrated, a wide range of nontrivial properties can be observed when screening the variable positions of a known consensus sequence, with additional complexity when using multiple binding sites.

### The advantages of multiple PRE combinations

Our library was based on our basic CARTIV promoter designed to regulate effector gene expression within the TME^10^, thanks to the abundance of inflammatory cytokines such as TNFα and IFNγ (36). TNFα and IFNγ might also be present in inflammatory sites other than TME. Therefore, additional PREs that correspond to TME and not to inflammatory sites will help^41^. Hypoxia is a hallmark of TME^42^, and we demonstrated the possible addition of an HCPRE to CARTIV^10^. The cumulative improvement of HCPRE with Seq 5 or Seq 130 (Fig. 6) suggests that even improved CARTIVs sustain modularity and optional enhancement by an additional third-party element.

## Conclusion

We present an approach to designing, screening, and functionally validating inducible promoters with different traits that may improve engineered immune cells. Yet, in order to evaluate the efficacy of treatments employing these promoters they should be coupled to an appropriate CAR and evaluated in an in-vivo model. The dogma of “one treatment fits all” is shifting towards precision treatment^43^. CARTIV promoters with variations and modular modifications can provide engineered immune cells for specific TMEs.

## Supplementary Information

Supplementary Figures.

Supplementary Table 1.

Supplementary Table 2.

## Data Availability

All high-throughput sequencing files were deposited at NCBI BioSample database under accession SAMN 21397074: CARTIV G1K1 Library (TaxID: 9606).
