# Supplementary material for: High throughput screen for the improvement of inducible promoters for tumor microenvironment cues
Source: Sci Rep. 2022 May 3;12:7169. doi: 10.1038/s41598-022-11021-1 (PMC9065017; doi:10.1038/s41598-022-11021-1)
Supplement: Supplementary file 1 — Supplementary Figures. [file 41598_2022_11021_MOESM1_ESM.pptx]

## Slide 1
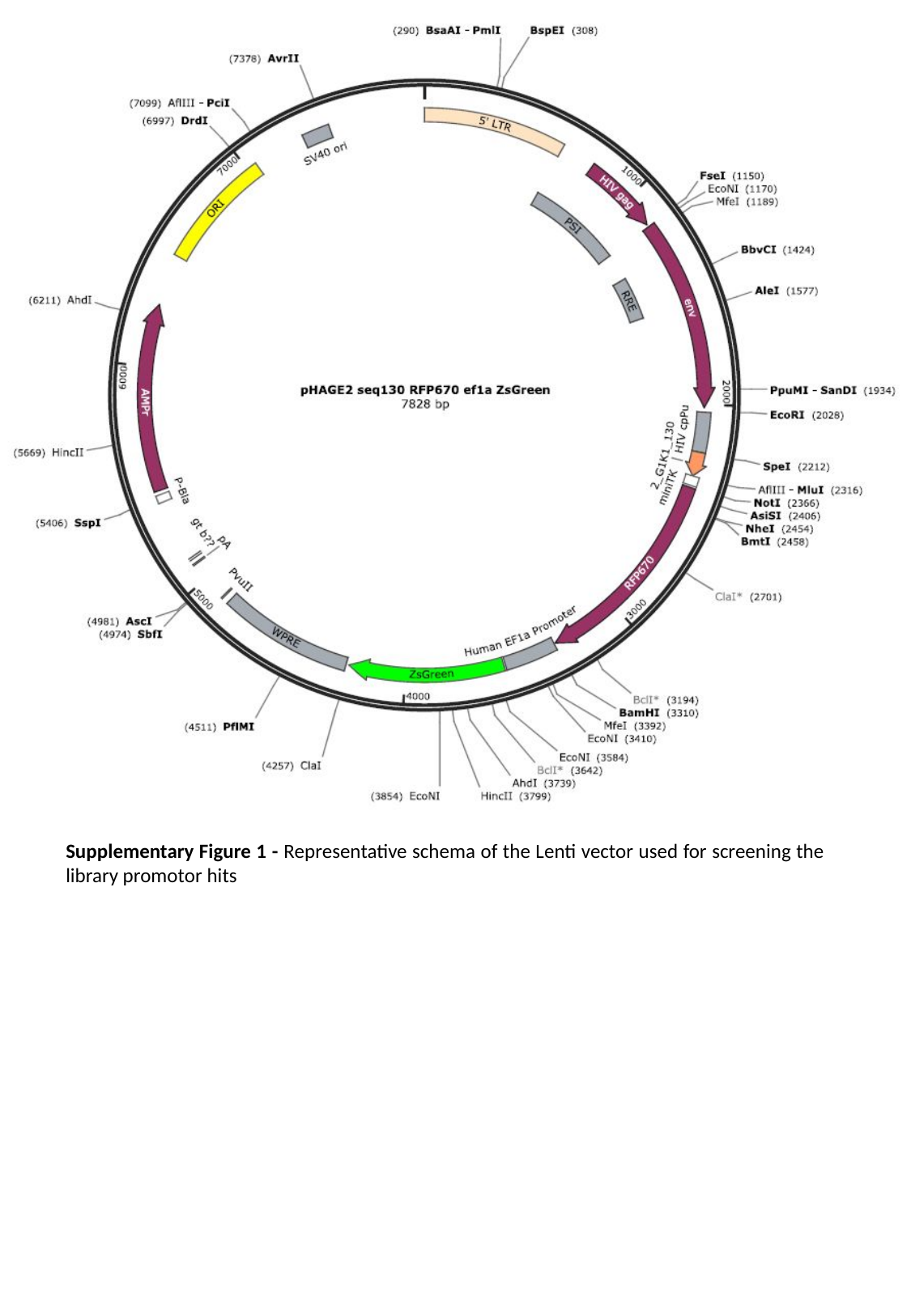

Supplementary Figure 1 - Representative schema of the Lenti vector used for screening the library promotor hits

## Slide 2
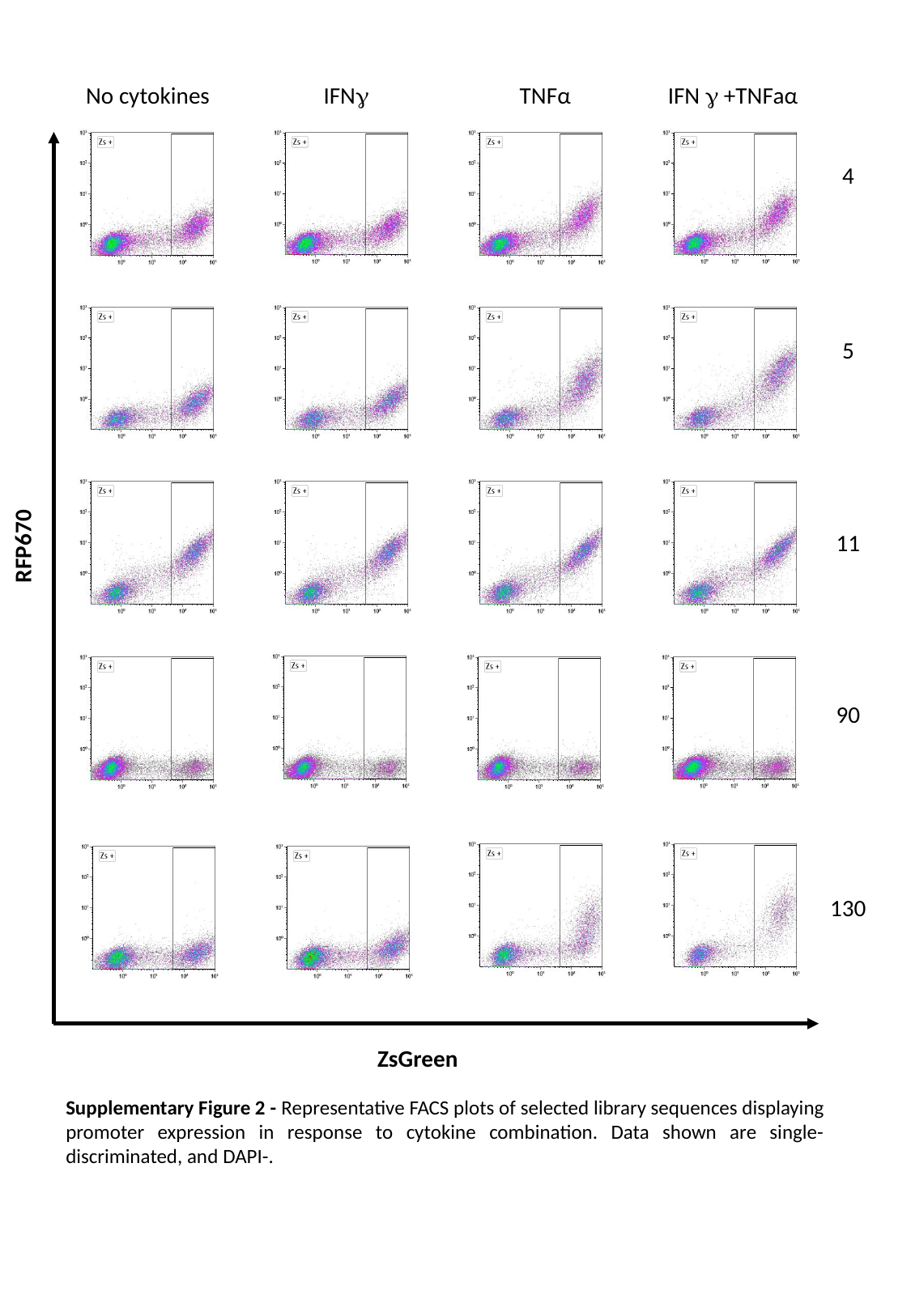

No cytokines
IFNγ
TNFα
IFN γ +TNFaα
4
5
11
RFP670
90
130
ZsGreen
Supplementary Figure 2 - Representative FACS plots of selected library sequences displaying promoter expression in response to cytokine combination. Data shown are single-discriminated, and DAPI-.

## Slide 3
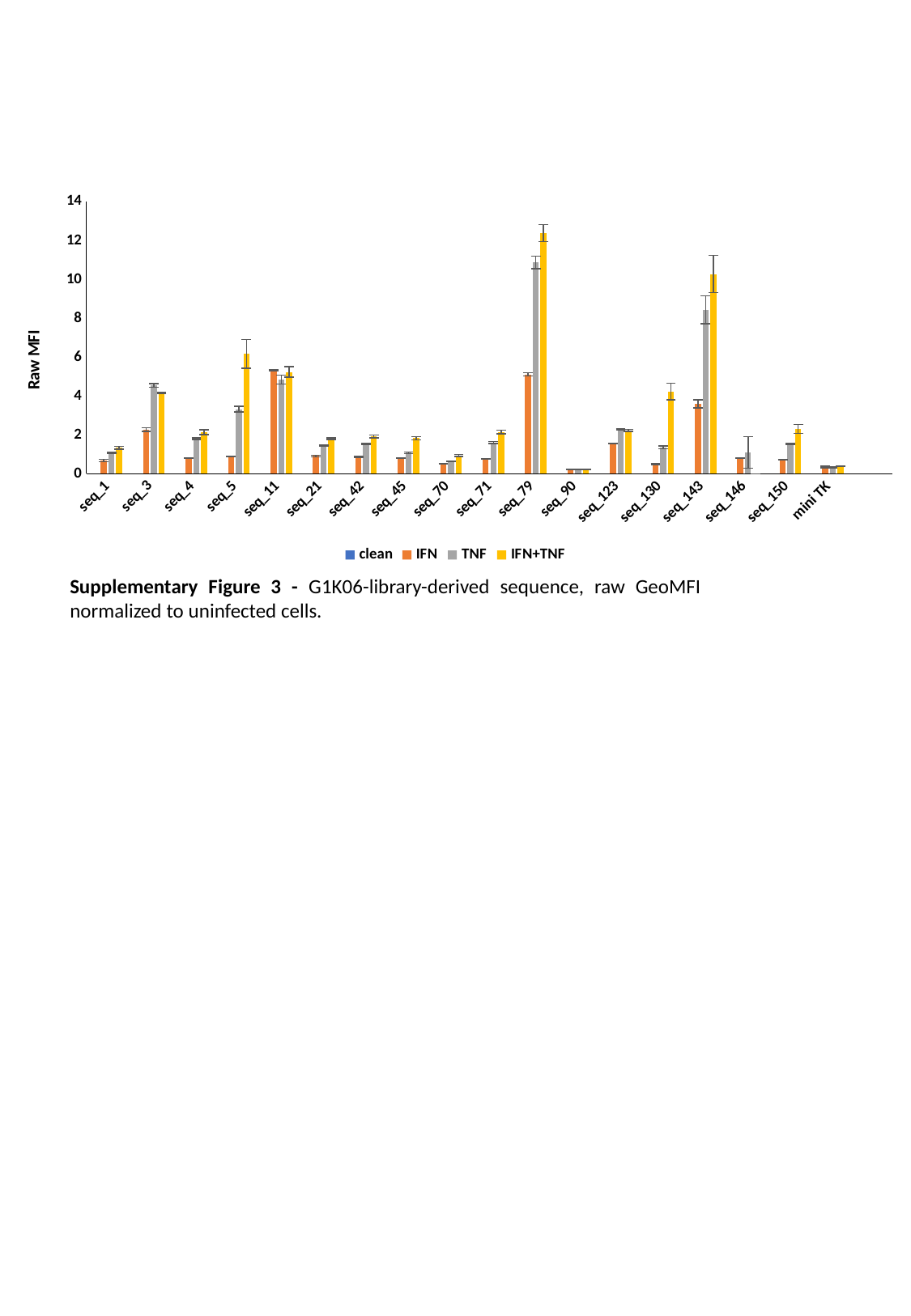

### Chart
| Category | clean | IFN | TNF | IFN+TNF |
|---|---|---|---|---|
| seq_1 | 0.6325 | 0.685 | 1.0875 | 1.3425 |
| seq_3 | 2.56 | 2.275 | 4.5425 | 4.155 |
| seq_4 | 0.8525 | 0.8125 | 1.8125 | 2.145 |
| seq_5 | 0.805 | 0.8975000000000001 | 3.32 | 6.170000000000001 |
| seq_11 | 4.8125 | 5.322500000000001 | 4.855 | 5.2475000000000005 |
| seq_21 | 1.0025 | 0.9175 | 1.4749999999999999 | 1.8075 |
| seq_42 | 0.84 | 0.87 | 1.5350000000000001 | 1.9224999999999999 |
| seq_45 | 0.7025 | 0.7975 | 1.0825 | 1.8325 |
| seq_70 | 0.4875 | 0.505 | 0.64 | 0.9475 |
| seq_71 | 0.7124999999999999 | 0.7549999999999999 | 1.6125 | 2.1550000000000002 |
| seq_79 | 5.8925 | 5.12 | 10.879999999999999 | 12.387500000000001 |
| seq_90 | 0.22749999999999998 | 0.22249999999999998 | 0.22749999999999998 | 0.23249999999999998 |
| seq_123 | 1.77 | 1.575 | 2.305 | 2.2424999999999997 |
| seq_130 | 0.35500000000000004 | 0.4925 | 1.3625000000000003 | 4.2325 |
| seq_143 | 3.9775 | 3.605 | 8.440000000000001 | 10.2825 |
| seq_146 | 1.31 | 0.81 | 1.105 | 0.0 |
| seq_150 | 0.76 | 0.7425 | 1.5375 | 2.3099999999999996 |
| mini TK | 0.32 | 0.35000000000000003 | 0.3275 | 0.37749999999999995 |Supplementary Figure 3 - G1K06-library-derived sequence, raw GeoMFI normalized to uninfected cells.

## Slide 4
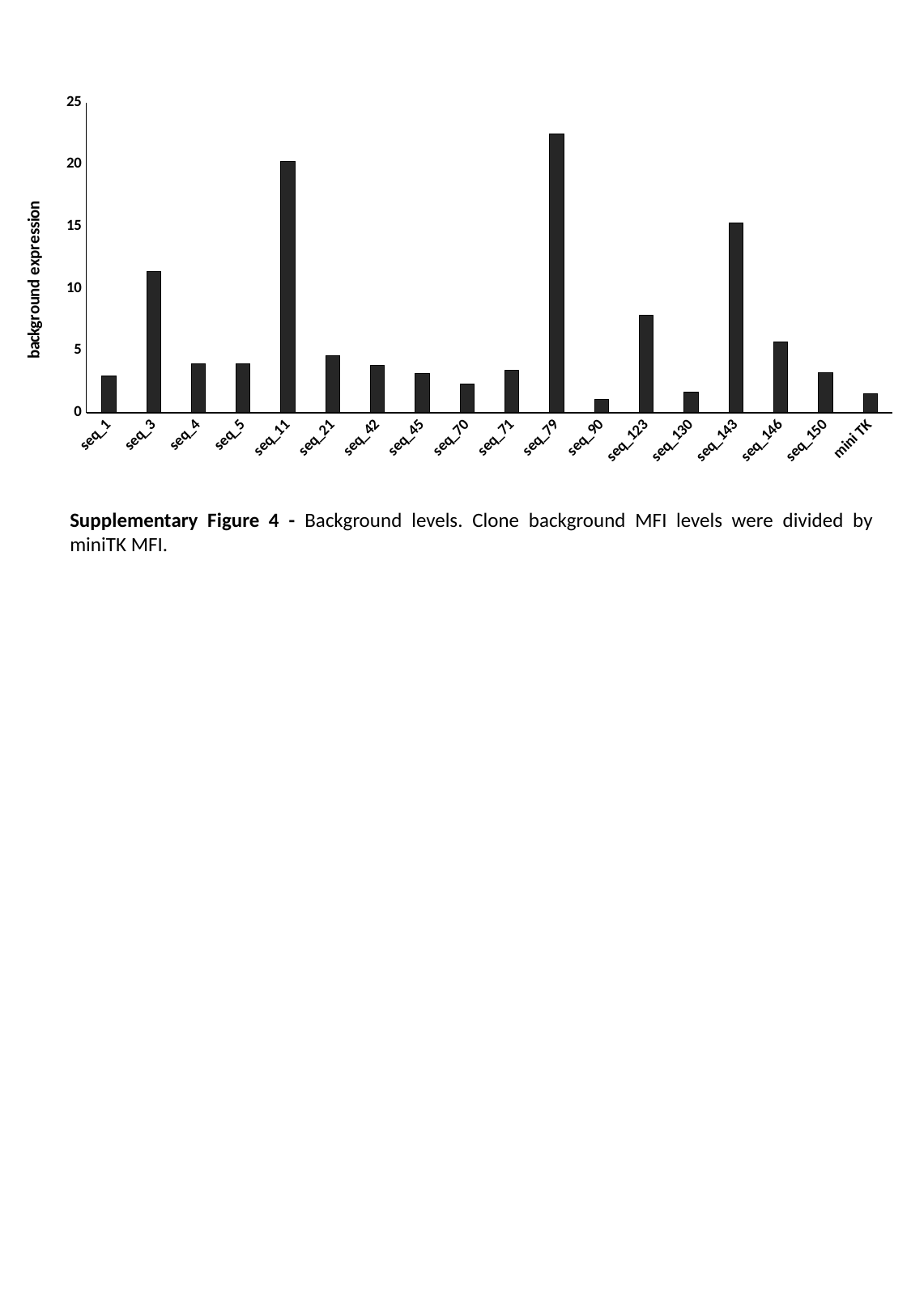

### Chart
| Category | |
|---|---|
| seq_1 | 2.9764705882352938 |
| seq_3 | 11.37777777777778 |
| seq_4 | 3.9195402298850577 |
| seq_5 | 3.926829268292683 |
| seq_11 | 20.263157894736842 |
| seq_21 | 4.5568181818181825 |
| seq_42 | 3.818181818181818 |
| seq_45 | 3.157303370786517 |
| seq_70 | 2.3214285714285716 |
| seq_71 | 3.3928571428571423 |
| seq_79 | 22.447619047619046 |
| seq_90 | 1.0459770114942528 |
| seq_123 | 7.866666666666666 |
| seq_130 | 1.6321839080459775 |
| seq_143 | 15.298076923076923 |
| seq_146 | 5.695652173913044 |
| seq_150 | 3.2 |
| mini TK | 1.5421686746987953 |Supplementary Figure 4 - Background levels. Clone background MFI levels were divided by miniTK MFI.

## Slide 5
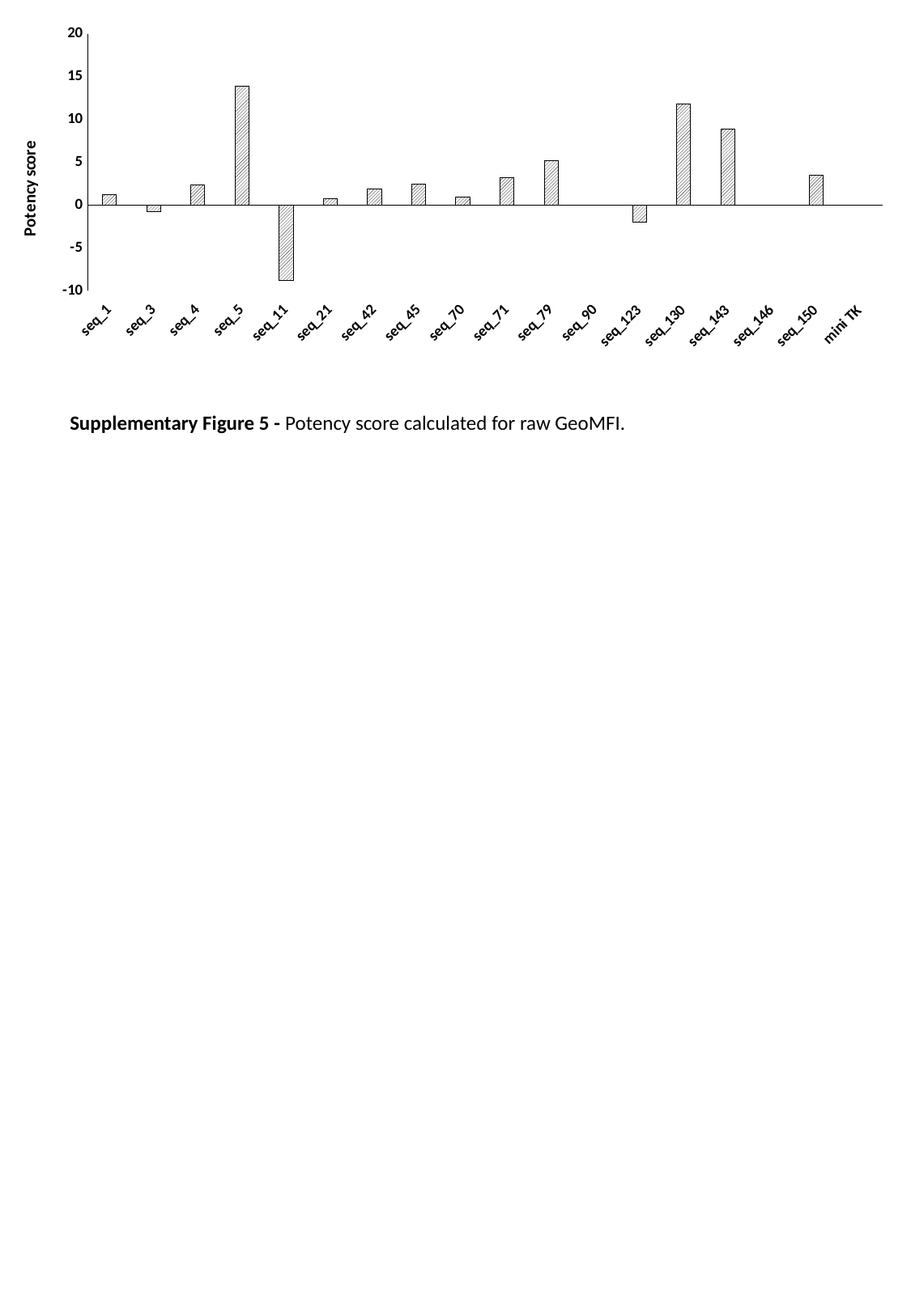

### Chart
| Category | |
|---|---|
| seq_1 | 1.2655034151307412 |
| seq_3 | -0.7212659349394597 |
| seq_4 | 2.390828327501112 |
| seq_5 | 13.927804774411563 |
| seq_11 | -8.776510699544898 |
| seq_21 | 0.7122400624107852 |
| seq_42 | 1.8438987600800418 |
| seq_45 | 2.4434965096400822 |
| seq_70 | 0.9880471015035592 |
| seq_71 | 3.185117203992233 |
| seq_79 | 5.19119792497806 |
| seq_90 | 0.03617516423309153 |
| seq_123 | -2.0117592134085616 |
| seq_130 | 11.782748315336999 |
| seq_143 | 8.923409620768048 |
| seq_146 | 0.0 |
| seq_150 | 3.469712590340448 |
| mini TK | 0.015026897256924188 |Supplementary Figure 5 - Potency score calculated for raw GeoMFI.

## Slide 6
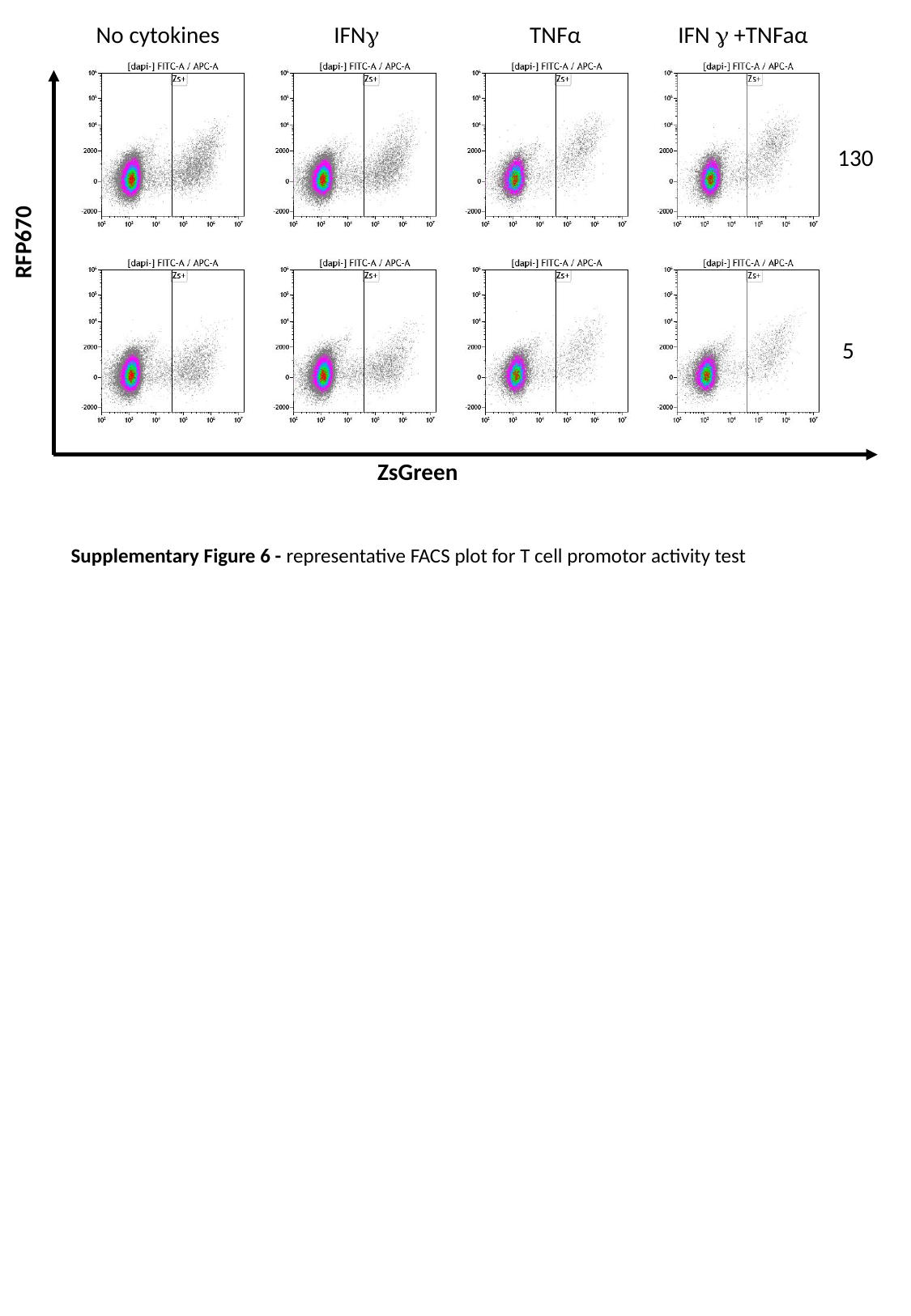

No cytokines
IFNγ
TNFα
IFN γ +TNFaα
130
RFP670
5
ZsGreen
Supplementary Figure 6 - representative FACS plot for T cell promotor activity test

## Slide 7
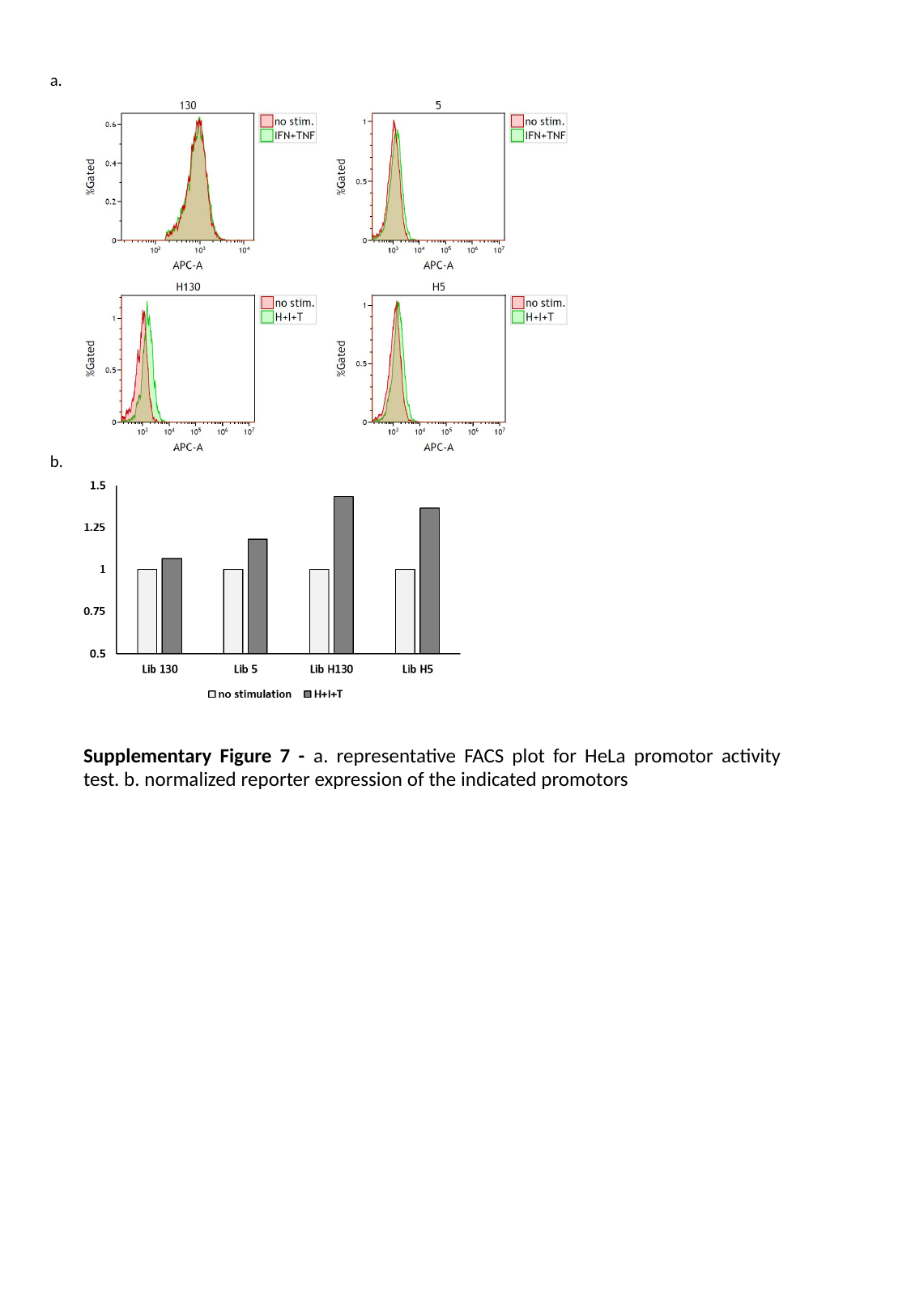

a.
b.
Supplementary Figure 7 - a. representative FACS plot for HeLa promotor activity test. b. normalized reporter expression of the indicated promotors
